# *Toxoplasma gondii* impairs CX3CL1/fractalkine shedding from mouse cortical neurons, leading to microglia activation

**DOI:** 10.1128/spectrum.01074-25

**Published:** 2025-08-13

**Authors:** Leonardo Leal de Castro, Barbara Gomes da Rosa, Maria Carolina Peixoto-Rodrigues, Amanda Roberta Revoredo Vicentino, Vanderlei da Silva Fraga-Junior, Cynthia M. Cascabulho, Luan Pereira Diniz, Claudia Farias Benjamim, Oliver Bracko, Julio Scharfstein, Daniel Adesse

**Affiliations:** 1 Laboratório de Biologia Estrutural, Instituto Oswaldo Cruz, Fiocruz 196605 , Rio de Janeiro, Brazil; 2 Laboratório de Imunologia Molecular e Celular, Instituto de Biofísica Carlos Chagas Filho, Universidade Federal do Rio de Janeiro 28125, Rio de Janeiro, Brazil; 3 Laboratory of Ocular Immunology and Transplantation, Bascom Palmer Eye Institute, University of Miami 5452, Miami, Florida, USA; 4 Laboratório de Inovações em Terapias, Ensino e Bioprodutos, Instituto Oswaldo Cruz, Fiocruz 196605 , Rio de Janeiro, Brazil; 5 Laboratório de Investigação Metabólica Associada ao Envelhecimento, Instituto de Ciências Biomédicas, Universidade Federal do Rio de Janeiro 28125, Rio de Janeiro, Brazil; 6 Department of Biology, University of Miami 124503, Miami, Florida, USA; 7 Department of Neurology, Leonard Miller School of Medicine, University of Miami 166788, Miami, Florida, USA; University of Illinois Urbana-Champaign, Urbana, Illinois, USA

**Keywords:** *Toxoplasma gondii*, blood-brain barrier, angiogenesis, neuroinflammation, fractalkine

## Abstract

Toxoplasmosis is caused by infection with *Toxoplasma gondii* and is one of the most prevalent food-borne parasitic disease worldwide. *T. gondii* disseminates through the host organism and forms a latency-specific structure called bradyzoite cysts, found primarily in muscle and neuronal cells. In mice, Toxoplasma leads to sustained brain microvascular abnormalities, including capillary rarefaction, microglial activation, and blood-brain barrier (BBB) breakdown, resulting in synaptic and neuronal loss, behavioral and cognitive damages. We hypothesized that cyst-bearing neurons could signal distinct classes of molecules that would orchestrate neurovascular and neuroinflammatory processes. Primary mouse cortical neurons were infected with *T. gondii* (ME49 strain) tachyzoites, which, 7 days post infection, generated cysts. We determined angiogenic-regulating factors from the neuronal conditioned media (nCM) using a proteome array and found nine molecules, belonging to four main functional clusters: (i) angiogenic signaling (VEGFA); (ii) endothelial-regulating growth factors (IGFBP-2, -3, -9 and PDGF-AA), (iii) chemoattractants (CCL-2, CCL-3, and CXCL12), and (iv) fractalkine signaling (CX3CL1). The main targets were validated in neuronal culture samples and in brain cortices by ELISA, RT-qPCR, or immunoblotting. CX3CL1 secretion was reduced in infected cultures and accumulated on neuronal surface. *In vivo*, the CX3CL1 receptor (CX3CR1) was upregulated, whereas the CX3CL1 soluble fraction was decreased. Recombinant CX3CL1 decreased arginase-1 and increased iNOS expression in nCM-treated microglial cells, indicating that CX3CL1 polarizes microglia to a pro-resolutive state. Our data suggest that CX3CL1 plays a key role in regulating neuroinflammatory signaling in acquired Toxoplasmosis, highlighting its potential to prevent the neurocognitive damage observed in infected individuals.

**IMPORTANCE:**

*Toxoplasma gondii* is a widespread parasite that forms latent cysts in neurons during chronic brain infection. How these infected neurons contribute to long-term brain damage is not well understood. In this study, we used a neuron-specific culture system and a mouse model to show that *T. gondii* infection alters the release of key signaling molecules by neurons. We found that infected neurons reduce secretion of fractalkine, a molecule that normally helps keep brain immune cells (microglia) in a resting state. At the same time, infected neurons showed increased expression of inflammatory and vascular-related genes, but not always matching increases in protein levels, pointing to complex regulation. These changes may contribute to blood-brain barrier dysfunction and persistent inflammation seen in chronic infection. Our findings highlight the role of neuron-derived signals in driving *T. gondii*-induced brain pathology and identify fractalkine as a potential target to reduce inflammation.

## INTRODUCTION

*Toxoplasma gondii* is an obligate intracellular parasite capable of infecting and replicating in the nucleated cells of warm-blooded animals (1). This parasite displays a global prevalence of over 30% of the human population, reaching up to 90% in some regions. Toxoplasmosis is considered the main food-borne disease although still a neglected disease (2). *T. gondii*’s life cycle involves a definitive host (felids) and intermediate hosts, that include mammals and birds (3). The parasite has three major evolutive forms: sporozoite, found in oocysts; tachyzoite found within parasitophorous vacuoles (PVs) in infected cells, and bradyzoite, found in tissue cysts. Tachyzoites replicate rapidly in nucleated cells until promoting cell egress and host cell lysis (4). Tachyzoites are released and disseminate through the organism, infecting new host cells, as characteristic of a rapid acute phase (5). Tachyzoites remain in a lytic cycle until immunological responses from the host signal tachyzoite-bradyzoite stage conversion. Bradyzoites are found in the chronic phase of the disease and are usually associated with infection latency. Following parasite differentiation, the PV undergoes morphological and structural alterations that give rise to a cyst (6). Tissue cysts can develop in several organs although they are consistently found in muscle and nervous tissues (7).

*T. gondii* infection in immunocompetent patients is usually asymptomatic, presenting symptoms such as fever and lymphadenopathy in the initial phase. Toxoplasmosis can present severe manifestations in cases of congenital disease or reactivation of latent infection in immunocompromised patients, including the elderly and patients with immunological syndromes, thus leading to ocular or cerebral toxoplasmosis. *T. gondii* forms cysts in the CNS, with this process occurring mainly in neurons (8). Our group has demonstrated that acquired infection in mice leads to cerebral microvascular dysfunction, with blood-brain barrier (BBB) breakdown as a consequence of a neuroinflammatory process, including an increase in leukocyte-endothelium interaction and microglial activation (9), contributing to cognitive dysfunction (10).

The brain microvasculature is composed primarily by specialized endothelial cells (EC) and protects the CNS from systemic circulating toxic compounds or molecules, immune cells, or pathogens (11–14) thus named BBB. BBB-forming ECs are connected by tight junction (TJ) proteins at the luminal side and display low transcytosis rates, as well as expression of membrane efflux pumps, that further confers a highly selective permeability. At the abluminal side, ECs interact closely with pericytes within the basement membrane onto which astrocytes project their endfeet (11–14). Combined with the input from neurons and astrocytic endfeet, this structure forms the Neurovascular Unit (NVU), along with signals from pericytes, smooth muscle cells, and microglia.

Given the fact that, in the CNS, *T. gondii* cysts are found in its entirety in neurons (15) and that brain microvascular abnormalities were directly associated with parasitism in mice (9, 16, 17), we hypothesized that cyst-bearing neurons could contribute to such abnormalities in chronic toxoplasmosis. In the present work, we determined angiogenesis-related factors differentially secreted from primary mouse cortical neuron cultures. Although neural stem cells are known to produce pro-angiogenic factors (18), mature neurons can also produce VEGF upon hypoxic stimulus (19–21). In this study, we established primary cortical neuron cultures and infected them with tachyzoites of *T. gondii* at a low multiplicity of infection (MOI) in order to allow formation of bradyzoite cysts. The secretome of infected and non-infected cultures was subjected to an angiogenic-related antibody array, in which we identified molecules that are potentially involved in immunoregulation and in EC biology. We further validated these targets in primary neuronal cultures and in an experimental model of acquired toxoplasmosis that leads to a brain microvasculopathy (9) and highlighted CX3CL1 (fractalkine) as an important player in Toxoplasma-induced neuropathology.

## MATERIALS AND METHODS

### Animals and parasites

Swiss Webster (SW) and C57BL/6 mice were obtained from the Instituto de Ciência e Tecnologia em Biomodelos (ICTB, Fiocruz) and kept at the Animal Facility of the Instituto Oswaldo Cruz with food and water *ad libitum* at 20°C. Pregnant SW female mice at E15-16 were euthanized immediately for the neuron preparations. For parasite maintenance, C57BL/6 mice received 50 cysts of *T. gondii*, ME49 strain (type II), via intraperitoneal route in 100 µL of 1× PBS. After 45 days of infection, animals were euthanized with a lethal dose of xylazine (35 mg/kg) and ketamine (350 mg/kg) diluted in saline solution, via IP. Death was verified by the absence of pedal reflex and confirmed with cervical dislocation. Brains were surgically removed and chopped in 1× PBS solution using sterile scissors. Brain suspensions were passed through different needles using sterile syringe with caliber up to 26 G. Cysts were counted in 20 µL of suspension and kept at 4°C for up to a month. To obtain free bradyzoites, cysts were ruptured with acid pepsin solution and free parasites were added to uninfected cultures of Vero cells (ATCC). After 2 weeks of culture re-infections, tachyzoites released from the supernatant were collected and centrifuged prior to use.

The use of mice in this work was approved by the Committee of Ethics in the Use of Laboratory Animals at the Oswaldo Cruz Institute (CEUA-IOC) under the license number L-038/2020.

### Primary mouse cortical neuron culture and experimental design

At gestational days 15–16, pregnant female SW mice were euthanized by a lethal dose of xylazine (35 mg/kg) and ketamine (350 mg/kg) diluted in saline solution, via IP. Death was verified by the absence of pedal reflex and confirmed with cervical dislocation. Embryos were surgically removed from the uterus and euthanized by decapitation. Fetal brain cortices were surgically dissected and mechanically dissociated in neurobasal medium, and plated at a density of 10^6^ cells per well in 6-well plates, which were previously coated with 10 µg/mL Poly-L-lysine (P9155, Sigma) diluted in water. Cells were kept with neurobasal medium supplemented with B-27 (ThermoFisher), penicillin/streptomycin solution (ThermoFisher), Glutamax (ThermoFisher), and 0.65 µM Cytarabine (AraC, SigmaAldrich). One week after plating, cells were infected by adding 10^5^ ME49 strain tachyzoites in neuronal maintenance medium. Infections were followed without replacing cell culture media and disturbing the dishes for seven days, until subsequent analyses.

### BV-2 mouse microglial cell line

Mouse microglial cell line BV-2 was obtained from the Rio de Janeiro Cell Bank (Banco de Células do Rio de Janeiro, reference #0356). Cells were maintained in RPMI medium supplemented with 10% FBS (Cultilab, Sao Paulo, Brazil) and 1% antibiotic solution (ThermoFisher) at 37°C with 5% CO_2_ atmosphere. For treatments with neuronal conditioned media (nCM), 350,000 cells were plated onto 6-well plates at a density of 36,458 cells/cm^2^ in maintenance medium, and after 24 h were treated with a 1:1 mixture of fresh maintenance medium and nCM (either from uninfected or *T. gondii*-infected cultures). An additional negative control (not shown) was made by incubating cells with fresh neuronal maintenance media with BV-2 media. Lipopolysaccharides from *Salmonella enterica* (LPS, #L6511, SigmaAldrich) were used as a positive control of microglial activation. LPS was diluted in RPMI medium and used at 1 µg/mL. Recombinant mouse fractalkine (rFKN, full length protein, 472-FF-025/CF, R&D) was used at 200 ng/mL. Twenty-four hours after treatment, microglial conditioned media was collected, centrifuged for 5 min at 10,000 rpm at 4°C, and stored at −80°C for nitrite determination, and monolayers were washed in ice-cold PBS and scraped in the presence of Trizol reagent for RNA extraction.

### Immunocytochemistry

One million cells were seeded onto 35 mm plates coated with 10 µg/mL Poly-L-lysine (Sigma P9115). Cultures were fixed at desired times with 4% paraformaldehyde in PBS for 10 min at 20°C, permeabilized with 0.5% Triton x-100 (Sigma-Aldrich, St. Louis, MO), blocked with 3% bovine serum albumin (BSA, Sigma-Aldrich , St. Louis, MO), and incubated overnight with primary antibodies at 4°C. Cells were washed with PBS and incubated with fluorescently labeled secondary anti-bodies for 1 h at 37°C. For nuclear visualization, cells were incubated with DAPI (4′,6-diamidino-2-phenylindole) and mounted in a solution of glycerol and DABCO (1,4-diazabicyclo [2.2.2] octane, Sigma-Aldrich, St. Louis, MO) in PBS. The list of primary and secondary antibodies used in this study is detailed in Table 1. F-actin filaments were stained with AlexaFluor 488 Phalloidin (ThermoFisher, reference number A12379) diluted in PBS 1:100 for 30 min at 37°C. *T. gondii* cysts were alternatively detected using fluorescein-labeled Dolichos Biflorus Agglutinin (DBA) lectin (Vector Laboratories).

**TABLE 1:** Primary antibodies used in this study^*a*^

Target protein	Host animal	Dilution	Manufacturer	Reference code
ADAM17	Rabbit	1:1,000 (WB)	ThermoFisher	703077
CX3CL1	Rabbit	1:200 (ICC); 1:1,000 (WB)	ThermoFisher	PA129224
GFAP	Mouse	1:200 (ICC)	ABCAM	ab7260
IL-1β	Rabbit	1:1,000 (WB)	Cell Signaling Technologies	#12703
MCP-1 (CCL2)	Rabbit	1:1,000 (WB)	Cell Signaling Technologies	#2029
NF-200	Rabbit	1:200 (ICC)	SigmaAldrich	N4142
SAG1 (P30), *T. gondii* tachyzoites	Mouse	1:100 (ICC)	ThermoFisher	MA183499
SDF-1 (CXCL12)	Rabbit	1:1,000 (WB)	ABCAM	ab9797
β3-Tubulin (TU-20)	Mouse	1:200 (ICC)	Cell Signaling Technologies	4466S

^*a*^ ICC, immunocytochemistry; WB, western blotting.

### Angiogenesis-related protein secretome

For the generation of nCM, cells were plated on 6-well plates and, after infection, were maintained in a total volume of 1 mL per well. Conditioned culture media were collected at 7 days post-infection (dpi) as described above and centrifuged for 5 min at 10,000 rpm at 4°C and stored at −80°C until use. Secretion of angiogenesis-related protein levels was detected using a Proteome Profiler Mouse Angiogenesis Antibody Array kit (R&D Systems) according to the manufacturer’s instructions. Membranes were incubated with nCM of two independent experiments as follows: Membrane 1: Mock culture, experiment #1; Membrane 2: *T. gondii*-infected culture #1; Membrane 3: Mock culture, experiment #2; Membrane 4: *T. gondii*-infected culture #2. Spots were developed with chemoluminescence, and X-ray films were exposed for 1, 5, 10, and 15 min to detect differentially expressed proteins. Densitometric analysis was performed with UN-SCAN-IT gel analysis software version 7.1, and relative intensity values for each spot of the 1 min exposed film were analyzed via GraphPad Prism software version 9.0.1.

### Cytokine profiling

Cytokine levels were evaluated by flow cytometry in culture supernatants of infected or uninfected neuronal cells at 7 dpi. IL-2, IL-4, IL-6, IL-10, IL-17A, TNF-α, and IFN-γ were detected using a Cytometric Bead Array (CBA) Mouse TH1/TH2/TH17 kit (BD) according to the manufacturer’s instructions. Data were acquired using a Cytoflex S flow cytometer (Beckman Coulter), and the data analysis was performed by a CBA analysis using the FCAP software version 3 (BD Biosciences). Values obtained were subtracted from baseline levels detected in fresh media samples.

### Enzyme-linked immunosorbent assay

nCM was collected and stored at −80°C as described above. ELISA for mouse IL-1β, CCL2/MCP-1, and CX3CL1/Fractalkine was performed with kits from R&D (reference numbers DY401, DY479, and DY472, respectively). nCM were applied directly to the wells and incubated overnight at 4°C. All procedures were performed according to manufacturer’s instructions, and plates were read by spectrophotometry at 562 nm.

### Nitrite measurement

Nitrite was measured in BV-2 microglia cell conditioned medium with the Griess reagent. Briefly, 50 µL of conditioned medium samples was assayed in triplicates to 50 µL of a 1% sulfanilamide solution in 5% H_2_PO_4_, followed by the addition of 50 µL of a 0.1% N-(1-Naphthyl)ethylenediamine aqueous solution. Each step had a 10 min incubation period at 20°C protected from light. A standard curve (1.56–100 µM) of nitrite was prepared also in triplicates with a 0.1 M sodium nitrite solution. Absorbance was read at 548 nm and nitrite concentration in biological replicas determined as a function of the standard curve.

### Acquired toxoplasmosis mouse model

For this study, we utilized a mouse model of *T. gondii* infection as described previously by our group (9). Twenty-day old SW female mice were inoculated *via* the intraperitoneal route with 50 *T. gondii* (Me49 strain) tissue cysts, isolated from previously infected C57BL/6 mice. This model is known to induce BBB leakage at 40 dpi, along with increased microglial reactivity and microvascular abnormalities. At desired times (10 and 40 dpi), mice were euthanized by lethal dose of xylazine (35 mg/kg) and ketamine (350 mg/kg) diluted in saline solution via IP. A minimum of six mice per experimental group (control or infected, for each time point) was utilized, divided into two independent rounds of infections. The brains were dissected in a way that one hemisphere was used for RT-qPCR and the other for western blotting.

### Western blotting

Brain cortices were dissected at desired infection times and lysed in the presence of RIPA Buffer (Santa Cruz Biotechnology). Protein concentration was measured with BCA Protein Assay Kit according to the manufacturer’s instructions (Thermo Fisher Scientific, Carlsbad, CA, USA). Then, 30 µg of protein was loaded onto a 10% Bis-acrylamide gel and resolved by electrophoresis at 100 V. Proteins were electrophoretically transferred to nitrocellulose membranes. Membranes were blocked with bovine serum albumin (BSA) 5% in TBS-0.05% Tween20 (TBST) and incubated overnight at 4°C with the primary antibodies at 1:1,000 dilution in TBST (Table 1). The next day, blots were washed with TBST, incubated for 1 h at room temperature with secondary antibodies (Lincoln, NE, USA), and analyzed using the Li-Cor CLX imaging system and the Image Studio 4.0 software (LI-COR, NE, USA).

### RT-qPCR

Cells were grown in 6-well plates, and total RNA from two wells per experimental condition was extracted with Trizol reagent (Thermo Fisher, Carlsbad, CA) according to the manufacturer’s instructions. Brains were dissected and cortices from one hemisphere per mouse had their RNA extracted with Trizol reagent with a TissueLyser (Qiagen). One microgram of total RNA was reversely transcribed into cDNA *via* the SuperScript III system (Thermo Fisher, Carlsbad, CA), and 0.5 µL of cDNA was used per RT-qPCR reaction with Power SYBR Green (Thermo Fisher, Carlsbad, CA) master mix. Reactions were read in QuantStudio V or 7500 StepOne Plus Real-time PCR cyclers (ThermoFisher). Primer sequences used in this study had efficiency between 90% and 110% (22), and their sequences are provided in Table 2. Gene expression variations were assessed by the 2^ΔΔCt^ method, with Ct as the cycle number at the threshold. Desired PCR result specificity was determined based on melting curve evaluation.

**TABLE 2:** RT-qPCR primer sequences used in this study

Target gene	Gene symbol	Forward primer (5’−3’)	Reverse primer (5′−3′)	Final concentration
Disintegrin and metalloproteinase domain-containing protein 10	*Adam10*	AGCAACATCTGGGGACAAAC	TTGCACTGGTCACTGTAGCC	250 nM
Disintegrin and metalloproteinase domain-containing protein 17	*Adam17*	TGTGAGCGGTGACCACGAGAAT	TTCATCCACCCTGGAGTTGCCA	300 nM
A disintegrin and metalloproteinase with thrombospondin motifs 1	*Adamts1*	CACGATGATGCTAAGCACTG	GGCTTGTCCATCAAACATTC	250 nM
Arginase1	*Arg1*	CTTGCGAGACGTAGACCCTG	TCCATCACCTTGCCAATCCC	400 nM
Cathepsin S	*Ctss*	GCCATTCCTCCTTCTTCTTC	CTAGCAATTCCGCAGTGATT	300 nM
C-C motif chemokine ligand 2	*Ccl2*	TGC TAC TCA TTC ACC AGC AA	GTC TGG ACC CAT TCC TTC TT	500 nM
C-C motif chemokine ligand 2	*Ccl3*	ACTGCCTGCTGCTTCTCCTACA	ATGACACCTGGCTGGGAGCAAA	300 nM
C-C motif chemokine receptor 1	*Ccr1*	GCCAAAAGACTGCTGTAAGAGCC	GCTTTGAAGCCTCCTATGCTGC	500 nM
C-C motif chemokine receptor 1	*Ccr2*	GCTGTGTTTGCCTCTCTACCAG	CAAGTAGAGGCAGGATCAGGCT	250 nM
Collagen alpha-1(XVIII) chain	*Col18a1*	GTGACACTGGACCTCAAGGCTT	TTGTCTGAAGGAGGGTCCTGGT	500 nM
C-X-C motif chemokine ligand 12	*Cxcl12*	CTTCATCCCCATTCTCCTCA	GACTCTGCTCTGGTGGAAGG	500 nM
C-X3-C motif chemokine ligand 1	*Cx3cl1*	CCAGAGCTGGCAATAACC TA	GGCATACAGGGTACGATCTG	500 nM
C-X3-C motif chemokine receptor 1	*Cx3cr1*	CTGTTATTTGGGCGACATTG	AACAGATTTCCCACCAGACC	400 nM
cellular communication network factor 1	*Ccn1* (Cyr61/Igfbp10)	TTCTTTTCAACCCTCTGCAC	GACTGGTTCTGGGGATTTCT	500 nM
Endothelin-1	*Edn1*	CCAAGCAGGAAAAGAACTCA	TCAACTTTGCAACACGAAAA	250 nM
Insulin-like growth factor-binding protein 2	Igfbp2	CCCCCTGGAACATCTCTACT	GGGTTTACTGCACACTTTGG	300 nM
Insulin-like growth factor binding protein 2	Igfbp3	CCTCAATGTGCTGAGTCCCAGA	CTTGTCCACACACCAGCAGAAG	300 nM
Cellular communication network factor 3	Ccn3 (Igfbp9/NOV)	CCAGGTGGAGTTTCAGTGTC	ACCATTTGCCTCAACCAATA	500 nM
Interleukin 1 beta	*Il1b*	CCCAACTGGTACATCAGCAC	TCTGCTCATTCACGAAAAGG	500 nM
Interleukin 6	*Il6*	ACAAGTCGGAGGCTTAATTACACAT	AATCAGAATTGCCATTGCACAA	500 nM
Nitric oxide synthase 2, inducible	*Nos2* (iNOS)	TGACGCTCGGAACTGTAGCAC	TGATGGCCGACCTGATGTT	400 nM
Low-density lipoprotein receptor-related protein 1	*Lrp1*	CGAGAGCCTTTGTGCTGGATGA	CGGATGTCCTTCTCAATGAGGG	300 nM
Platelet derived growth factor, B polypeptide	*Pdgfb*	AATGCTGAGCGACCACTCCATC	TCGGGTCATGTTCAAGTCCAGC	500 nM
Platelet derived growth factor receptor, alpha polypeptide	*Pdgfra*	TGAAGCAAGCTGATACCACA	TCCTCGAGCAACTTGATAGG	500 nM
Transforming growth factor, beta 1	*Tgfb1*	GGTTCATGTCATGGATGGTGC	TGACGTCACTGGAGTTGTACGG	500 nM
Transmembrane protein 219	*Tmem219*	GAACTTCGGAGATGGTCCAGAC	CTGTGACAAGGACAGACTCTCC	150 nM
Thrombospondin 1	*Thbs1*	AAAGCCAAAGCGCCTATTTA	TGGCGGTGAGTTCTAGTGAG	500 nM
Vascular endothelial growth factor A	*Vegfa*	CTGCTGTAACGATGAAGCCCTG	GCTGTAGGAAGCTCATCTCTCC	500 nM
FMS-like tyrosine kinase 1	*Flt1* (VEGFR1)	GCAGTCTGAGAGGAGCTAAAG	GGAGAGGGAGCTTGCATAAA	500 nM
Kinase insert domain protein receptor	*Kdr* (VEGFR2)	GGCGAATCACTCACACCAGT	GCAATTCTGTCACCCAGGGAT	500 nM

### Statistical analyses

For RT-qPCR and ELISA, a minimum of five independent cell culture preparations we used and analyzed with Unpaired Student’s *T* test; at least six mouse cortices were used and analyzed with two-way ANOVA with Bonferroni post hoc-test, in GraphPad Prism Software v 10.3.0.507. Changes were considered statistically significant when *P* value was less than 0.05.

## RESULTS

### Characterization of the *in vitro* cystogenesis model in cortical neurons

**Fig 1:**
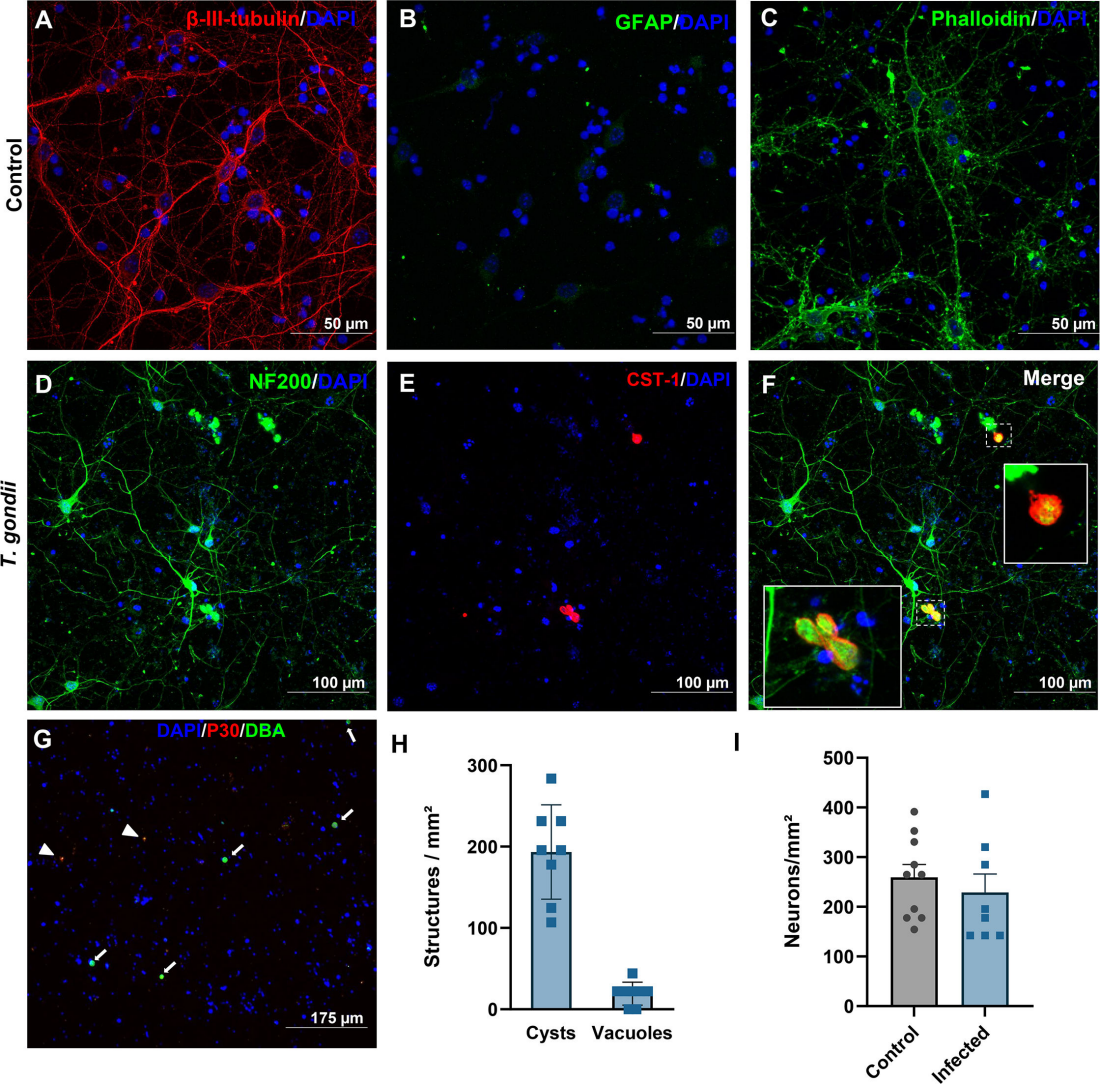
Establishment of an *in vitro* model of latently infected mouse cortical neuronal cultures. Primary neuronal cultures were established from E18 mouse cortices. After 14 days *in vitro*, neurons were detectable by β-III-tubulin staining (in red, in A), whereas no astrocytic cells were detected by GFAP staining (in green, in B). Overall neuronal morphology was visualized by Phalloidin staining (in green, in C). Cultures were infected with ME49 tachyzoites at 7 days *in vitro* and visualized 7 days after (14 days *in vitro*). Infection did not disturb neuronal morphology as depicted by neurofilament (NF200, green signal in D and F), and bradyzoite cysts were detected with anti-CST1 antibody (red signal in E). Merged image in F shows the presence of bradyzoite cysts within neurons. Insets in F show higher magnification of the cysts selected with the dashed squares. After 7 days of infection, few tachyzoites were detected in the cultures by SAG1 staining (red signal in G), as well as bradyzoite cysts stained with DBA lectin (green signal in G). Host cell nuclei were detected with DAPI (blue signal A–G). DBA-stained cysts were more abundant at this time point than tachyzoite vacuoles (**H**). Infection did not affect neuronal cellularity (**I**). Each point in (**H**) represents independent cultures, and in (**I**), microscopic fields from two independent cultures. Bars: 50 µm in (**A–C**); 100 µm in (**D–F**), and 175 µm in (**G**).

Neuronal cells were infected after 7 days *in vitro* (div) and infected with tachyzoites of the ME49 strain at a 0.1 MOI. Cultures showed an overall enrichment with neurons, as demonstrated by immunostaining for β-III-tubulin (Fig. 1A) with the absence of positive signal for glial marker GFAP (Fig. 1B); overall neuronal morphology was visualized with Phalloidin staining (Fig. 1C). Bradyzoite cysts were found within neurofilament-positive neurons (Fig. 1D through F) as detected by CST-1 immunostaining (Fig. 1E and F). Infected cultures displayed higher density of cyst bearing cells than those bearing tachyzoite-formed parasitophorous vacuoles (PV), as shown by SAG1/p30 staining (Fig. 1G). Neuronal cultures had 17 ± 588 cysts/mm^2^ and 19 ± 14 PV/mm^2^ (Fig. 1H). Neuronal cell counts, determined by the number of DAPI-stained neuronal nuclei, were not affected by long-term infection as shown in Fig. 1I. Additionally, no changes were found in the number of picnotic nuclei, an indicator of terminal apoptotic cell death (*not shown*).

### Angiogenic and inflammatory molecule secretion by neurons

To test whether *T. gondii*-infected neurons would produce a distinct secretory profile, nCM was initially subjected to CBA (Fig. S1). Overall cytokine production was low, ranging from 2 to 30 pg/mL in control cultures, consistent to what our group recently described for mouse neural progenitor cell primary cultures (23). No changes were observed for IL-2, -4, -6, -10, and -17A and IFN-γ; TNF had a 1.19-fold increase in infected cultures (*P* < 0.05, unpaired Student’s *T* test, Fig. S1).

**Fig 2:**
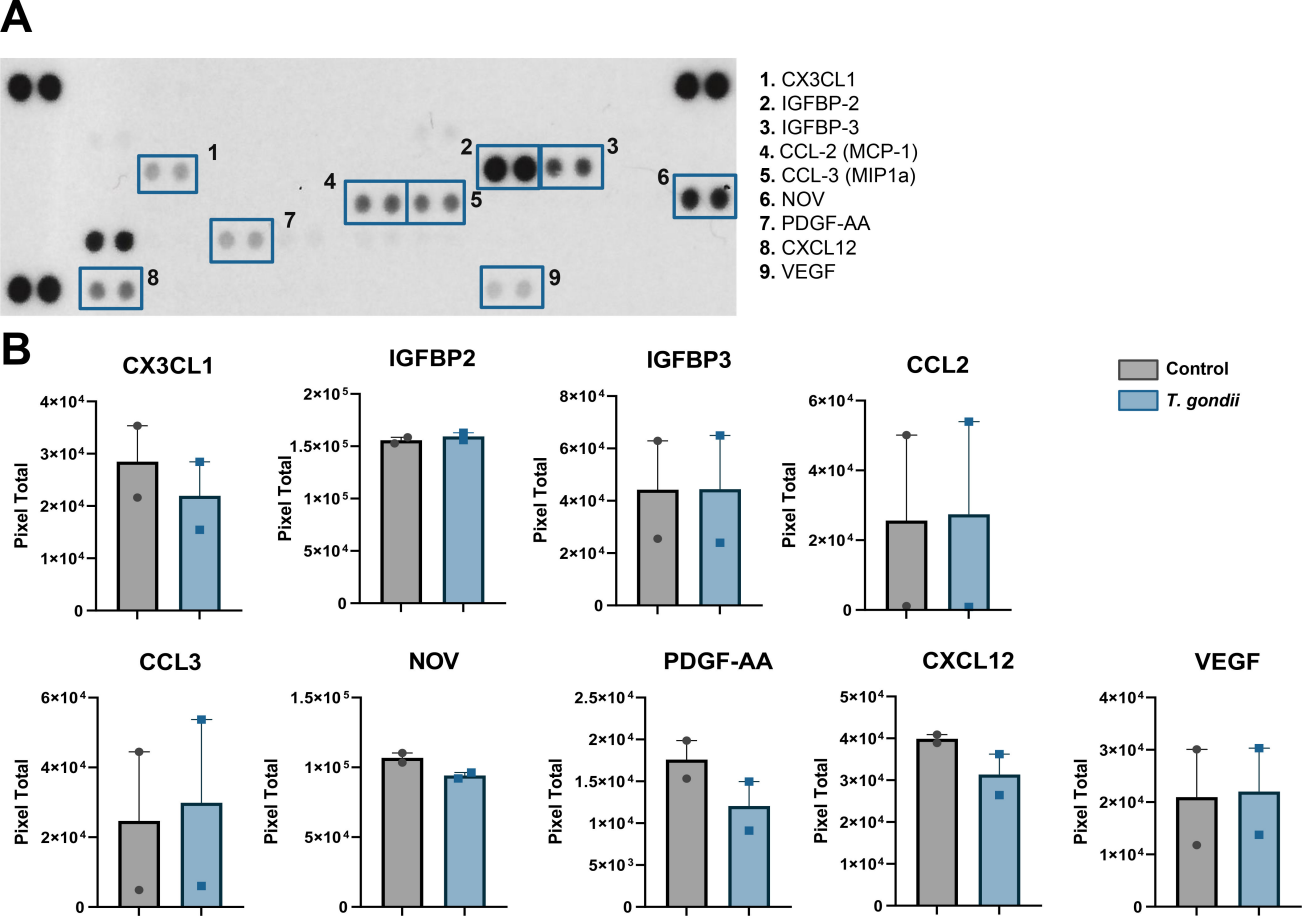
Characterization of neuron-derived angiogenic factors in latently infected cultures. Neuronal conditioned media (nCM) was collected at 7 dpi from two independent cultures and applied to angiogenic proteome array membranes. One representative array of uninfected culture is depicted in (**A**). Target proteins are spotted in duplicates, marked with blue squares, and their identities are revealed by the numbers written next to each square, as follows: CX3CL1 (1), IGFBP-2 (2), IGFBP-3 (3), CCL-2 (4), CCL-3 (5), NOV (6), PDGF-AA (7), CXCL12 (8), and VEGF (9). Densitometric analyses of two control and two infected cultures are shown in (**B**) for each detected analyte. Each dot in graphs represent the signal intensity in pixels from two spots average from each membrane, for each analyte.

In order to determine the angiogenesis-related secretory repertoire in cortical neurons, nCM from *T. gondii* infected and uninfected cultures were applied onto membranes of Proteome Profiler Mouse Angiogenesis Antibody Array kit, as shown in Fig. 2A. Out of the 55 spotted targets, 20 had detectable signals and 13 had positive signals in both experimental replicas (Fig. 2A and Table 3). Insulin-like growth factor-binding protein 2 (IGFBP2) had consistently the highest levels in nCMs, ranging from 152,824 to 162,839 pixel densities (Fig. 2B) although no significant change was observed in infected cultures. Other two members of the IGFBP family, IGFBP3 and IGFBP9, were also detected at high levels in nCM although no significant changes in pixel densities were observed in infected cultures as compared to control. IGFPB9 is encoded by the *nov* (nephroblastoma overexpressed) gene, which belongs to the CCN protein family, that includes Cyr61 (cysteine-rich angiogenic protein 61) and CTGF (connective tissue growth factor, or CCN2) (24). Cyr61 was increased in infected cultures in one of the two membrane assays in this study (Table 3). Platelet-derived growth factor AA (PDGF-AA) and PDGF-AB/PDGF-BB were detected in nCMs and had pixel signals equally reduced in infected cultures by 67% and 70%, respectively (Fig. 2B). Other analytes detected in nCM from both assays were endostatin, CCL-2/MCP-1, CCL3/MIP-1α, MMP-3 (pro- and mature forms), osteopontin, SDF-1/CXCL12, VEGF/VPF, and CX3CL1/Fractalkine. We found that ADAMTS1, Cyr61, endothelin, IL-1α and IL-1β, pentraxin, and CXCL4 (platelet factor 4) were present in only one of the two assays (Fig. 2 and Table 3). Fractalkine signal was consistently reduced in both experiments, by 29% and 20% (Fig. 2).

**TABLE 3:** Mini-proteome complete data^*a*^

Analyte	Control #1	Infected #1	Fold-change (%)	Control #2	Infected #2	Fold-change (%)	Averaged fold-change
ADAMTS1/METH1	–^*b*^	315.00	–	15,065.00	11,199.00	–	–
Cyr61/CCN1, IGFBP-10	–	185.50	–	2,506.00	8,654.00	–	–
Endostatin/Collagen XVIII	1,983.50	7,345.00	370.31	2,069.00	1,177.50	56.91	213.6083
Endothelin-1	–	–	–	–	190.50	–	–
**Fractalkine/CX3CL1**	**21,633.00**	**15,447.00**	**71.4**	**35,364.50**	**28,439.50**	**80.42**	**75.9115**
**IGFBP-3**	**62,914.50**	**64,976.50**	**103.28**	**25,527.50**	**23,939.00**	**93.78**	**98.52738**
**IGFBP-2**	**158,517.00**	**155,947.00**	**98.379**	**152,824.50**	**162,839.50**	**106.6**	**102.466**
IL-1α/IL-1F1	185.00	–	–	–	–	–	–
IL-1β/IL-1F2	150.00	–	–	186.00	–	–	–
MCP-1/CCL2/JE	50,118.50	53,969.50	107.68	1,135.50	860.00	75.74	91.71067
**MIP-1α/CCL3**	**44,452.50**	**53,719.00**	**120.85**	**4,893.50**	**6,047.50**	**123.6**	**122.2141**
MMP-3 (pro and mature form)	899.00	7,421.00	825.47	644.50	949.50	147.3	486.3981
**NOV/CCN3, IGFBP-9**	**103,398.00**	**92,029.00**	**89.01**	**110,262.00**	**96,290.00**	**87.33**	**88.17**
Osteopontin/OPN	98,820.00	84,783.50	85.80	908.50	256.00	28.18	56.99
**PDGF-AA**	**19,871.50**	**14,976.50**	**75.37**	**15,314.00**	**9,111.50**	**59.5**	**67.43**
PDGF-AB/PDGF-BB	1,018.50	842.00	82.67	6,483.00	3,790.50	58.47	70.57
Pentraxin-3/PTX3, TSG-14	541.00	755.00	139.56	–	–	–	–
Platelet Factor 4/CXCL4, PF4	–	–	–	349.00	120.00	34.38	–
**SDF-1/CXCL12**	**40,897.50**	**36,246.00**	**88.63**	**38,883.50**	**26,448.50**	**68.02**	**78.32315**
**VEGF/VPF**	**11,772.50**	**13,739.00**	**116.7**	**30,099.00**	**30,314.00**	**100.7**	**108.7092**

^*a*^ Analytes in bold indicate that pixel signal was higher than 5,000 in both biological replicas.

^*b*^ Empty cells (with the “–” symbol) represent spot in which signal was not detectable. In cases where such spots were absent in at least of membrane of each experiment (either Experiment #1 or #2), fold-change was not calculated, and “–” symbol was also utilized.

### Pro- and anti-angiogenic balance is disrupted by *T. gondii* infection

**Fig 3:**
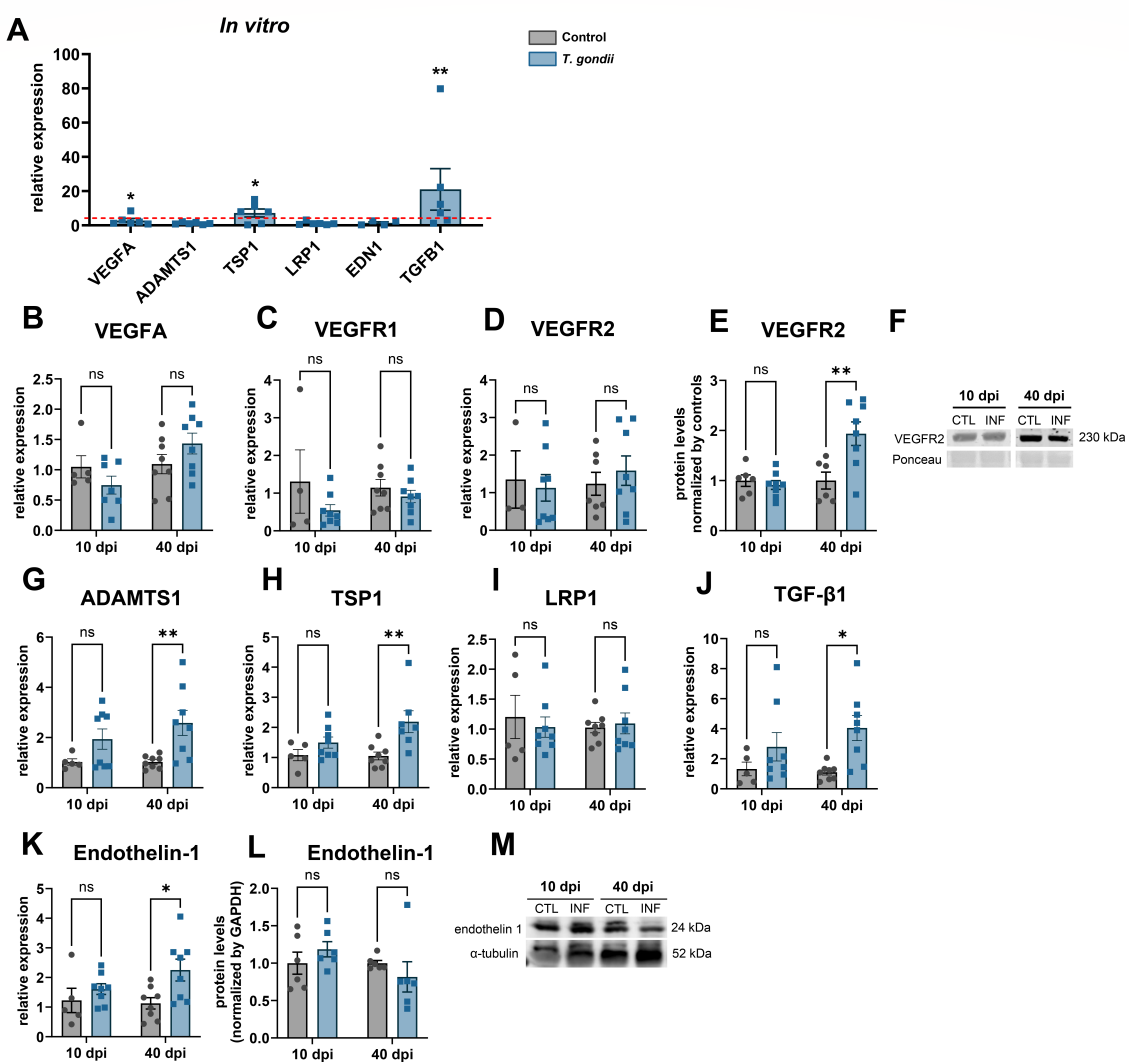
Angiogenic factors identified in neuronal cultures. Based on the mini proteome assays, three main regulators of endothelial cell biology were detected: VEGF, ADAMTS1 and endothelin-1. Neuronal cultures were analyzed by RT-qPCR at 7 dpi for *Vegfa*, *Adamts1*, *Tsp1*, *Lrp1*, *Edn1,* and *Tgfb1* expression (**A**). Data are shown as the relative expression, normalized by the controls (represented by the red dashed line). Mouse brain cortices were analyzed after 10 and 40 days of *T. gondii* infection (**B–M**) by RT-qPCR or western blotting. *Vegfa* (**B**), VEGFR1 (*Flt1*, **C**), VEGFR2 (*Flk1*, **D**) transcripts remained unaltered in infected brains, whereas VEGFR2 protein levels were increased at 40 dpi (**E and F**). *Adamts1* (**G**), *Tsp1* (**I**), *Tgfb1* (**J**), and *Edn1* (**K**) mRNA levels were increased at 40 dpi in infected cortices, and *Lrp1* (**I**) had no significant changes. No changes were found in endothelin-1 protein levels (**L and M**). (**F and M**) show representative blots for VEGFR2 and Endothelin-1, respectively. For VEGFR, signal from the strongest Ponceau-stained band signal was used as loading control, and for endothelin-1, α-tubulin was used. Each dot in (**A**) corresponds to independent neuronal cultures; in (**B–L**), dots represent independent mice from at least two rounds of infections. *P < 0.05, ** *P* < 0.01. Unpaired Student’s *T* test in (**B**) and two-way ANOVA with Bonferroni post-test in (**B–L**).

Angiogenesis is a process that accompanies repair and is associated with BBB breakdown after pathogenic stimuli such as brain injury or ischemia (25, 26), and is regulated by a finely tuned balance between expression of anti- and pro-angiogenic factors, that regulated endothelial cell (EC) proliferation and migration (27, 28). Vascular endothelial growth factor A (VEGFA) has a major role in angiogenesis in the brain after injury (29) and was shown to be transiently increased in response to trauma (26). Although VEGF/VPF was consistently secreted by cortical neurons and had a trend to be increased in *T. gondii*-infected cultures, no change was observed in *vegfa* gene expression *in vitro* (Fig. 3A) or in an *in vivo T. gondii* acquired mouse infection model (Fig. 3B), in which we previously demonstrated capillary rarefaction and reduced angiogenic parameters in brain endothelia (9). VEGF receptors (VEGFR1 and VEGFR2) had no change in mRNA levels in brain cortices, but VEGFR2 had a significant increase in protein levels in infected brains at 40 dpi, as compared to their respective controls (Fig. 3C through F). This could indicate that the pro-angiogenic effects of VEGF signaling pathway could be counterbalanced by increased expression of anti-angiogenic molecules that would then lead to capillary rarefaction observed in our model. Other factors that can directly affect BMEC biology and, thus, regulate angiogenic processes were also identified in our mini-proteome assay, such as ADAMTS1, endothelin-1, PDGFAA, and CCN family members (Fig. 2 and 3).

ADAMTS1 is a potent anti-angiogenic metalloprotease, which blocks VEGFR2 phosphorylation and suppresses endothelial cell proliferation (30). *Adamts1* expression was not significantly affected by *T. gondii* infection *in vitro* (Fig. 3A) but had a 1.94- (*P* > 0.05) and 2.58-fold change (*P* < 0.01) in mouse cortices at 10 and 40 dpi, respectively (Fig. 3G). Given that ADAMTS1 has a thrombospondin domain (31) and that thrombospondin1 (TSP1) is a matricellular protein with potent anti-angiogenic properties (32), we investigated TSP expression in neuronal cultures and mouse cortex. We found *tsp1* transcripts to be significantly increased in neuronal cultures after *T. gondii* infection by 7.21-fold (*P* < 0.05, Fig. 3A) and *in vivo* in mouse cortex at 10 (1.5-fold, *P* > 0.05) and 40 (2.2-fold, *P* < 0.01) dpi (Fig. 3H). TSP-1 can also interact with LDL receptor-related protein 1 (Lrp1) to remodel integrins and other focal adhesion molecules (33). We found no significant changes to Lrp1 expression in neuronal cultures (Fig. 3A) or in brain cortices (Fig. 3I). Transforming growth factor, a downstream target of thrombospondins (34) was found upregulated both *in vitro* (Fig. 3A) and *in vivo* in brain cortices (Fig. 3J).

Endothelin-1 (EDN1) is a potent vasoconstrictor that regulates vascular tonus through pericyte modulation, which, in turn, contributes to blood flow dysregulation and BBB breakdown (35, 36). Edn1 mRNA expression was not altered in *T. gondii*-infected neurons (Fig. 3A), but was increased in mouse brain cortices after 10 and 40 dpi by 1.6- (*P* > 0.05) and 2.25-fold (*P* < 0.05, Fig. 3K). However, no changes to endothelin-1 protein contents were observed in mouse cortices (Fig. 3L and M).

### Expression of endothelium modulators

**Fig 4:**
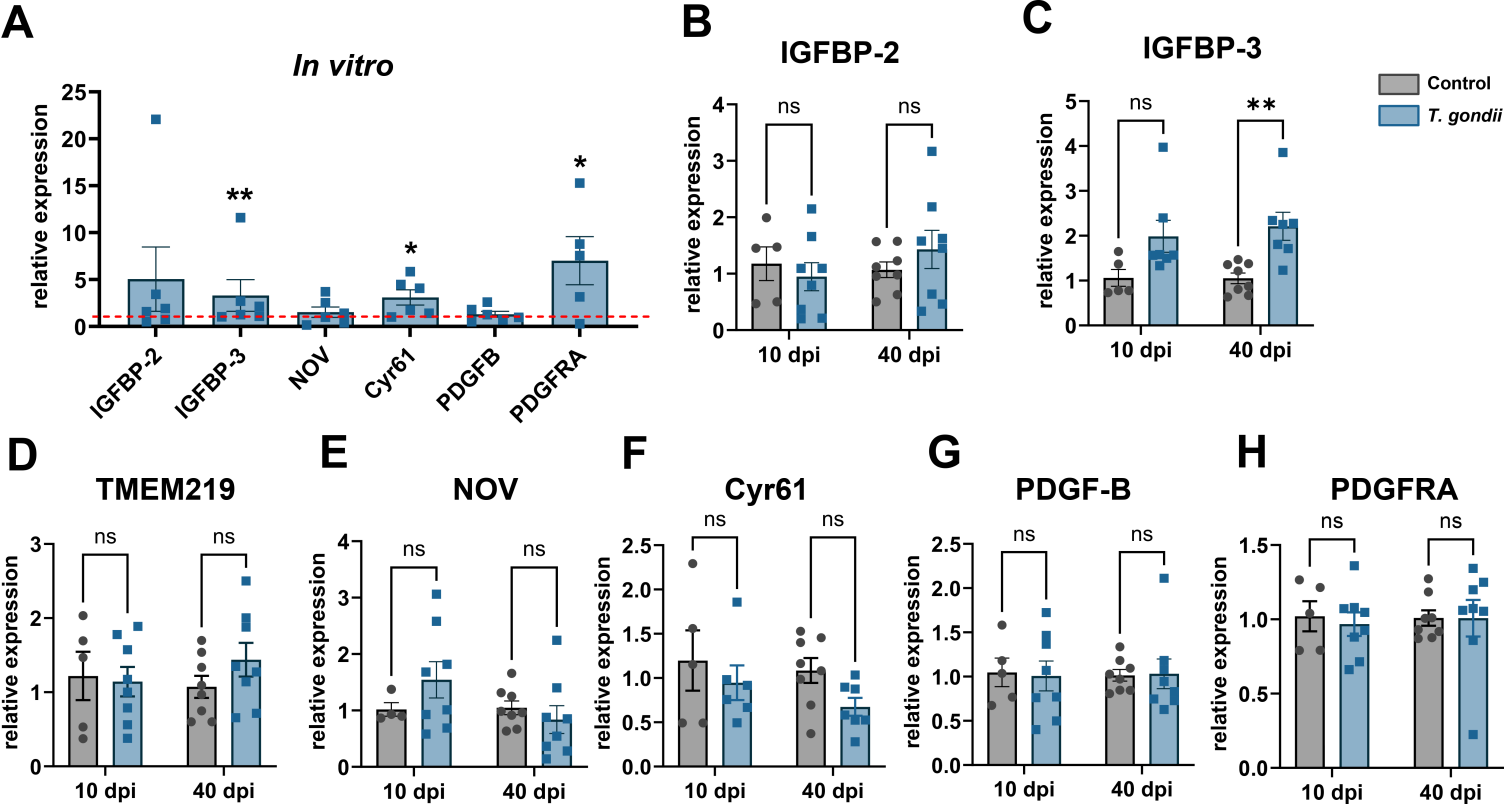
Endothelium-modulating growth factors released by *T. gondii*-infected neurons. Mini-proteome assays using nCM revealed differential secretion of matrikines IGFBP-2, IGFBP-3, and NOV, as well as of PDGF-AA. Neuronal cultures were infected with *T. gondii* ME49 strain tachyzoites, and mRNA was analyzed at 7 dpi for *Igfp2*, *Igfbp3*, *Ccn3* (NOV), *Ccn1* (Cyr61), *Pdgfb,* and *Pdgfra* by RT-qPCR (**A**). Data are shown as the relative expression, normalized by the controls (represented by the red dashed line). IGFBP3, Cyr61, and PDGFRA transcripts were significantly increased in infected cultures. Mouse brain cortices were analyzed after 10 and 40 days of *T. gondii* infection (**B–H**) by RT-qPCR for the same target genes, as well as for IGFBP3 receptor, TMEM219 (**D**). IGFBP3 was significantly increased in mouse cortices at 40 dpi. Each dot in (**A**) corresponds to independent neuronal cultures; in (**B–H**), dots represent independent mice from at least two rounds of infections. *P < 0.05, **P < 0.01. Unpaired Student’s *T* test in (**A**) and two-way ANOVA with Bonferroni post-test in (**B–H**).

Another class of proteins identified in nCMs were proteases related to extracellular matrix, IGF and TGF-β signaling pathways: insulin growth factor-binding proteins (IGFBPs) -2, -3, and -9 (NOV) as well as the platelet-derived growth factor beta (PDGF-B) (Fig. 4). These molecules are known regulators of the vascular endothelium, and we analyzed their gene expression levels. IGFBP3 and Cyr61 had a significant upregulation in *T. gondii*-infected neuronal cultures, as compared to uninfected controls (3.3- and 3.1-fold, respectively, Fig. 4A). Since neurons can also express the PDGF receptor alpha, we analyzed its expression and observed a sevenfold increase in infected cultures (Fig. 4A). No changes to IGFBP-2, NOV, and PDGF-B transcripts were found in *T. gondii*-infected cultures. The levels of IGFBP-2 and -3 were analyzed in mouse cortices of *T. gondii*-infected mice (Fig. 4B and C), and we found a significant upregulation of IGFBP-3 at 40 dpi (Fig. 4C). TMEM219, a known downstream component of the IGFBP-3 signaling pathway (37, 38), was not altered in infected mouse cortices (Fig. 4D). The expression of NOV, Cyr61, PDGF-B, and PDGFRA remains unaltered in *T. gondii*-infected brain cortices (Fig. 4E through H).

### Latent *T. gondii* infection modulates chemotaxis in cortical neurons

**Fig 5:**
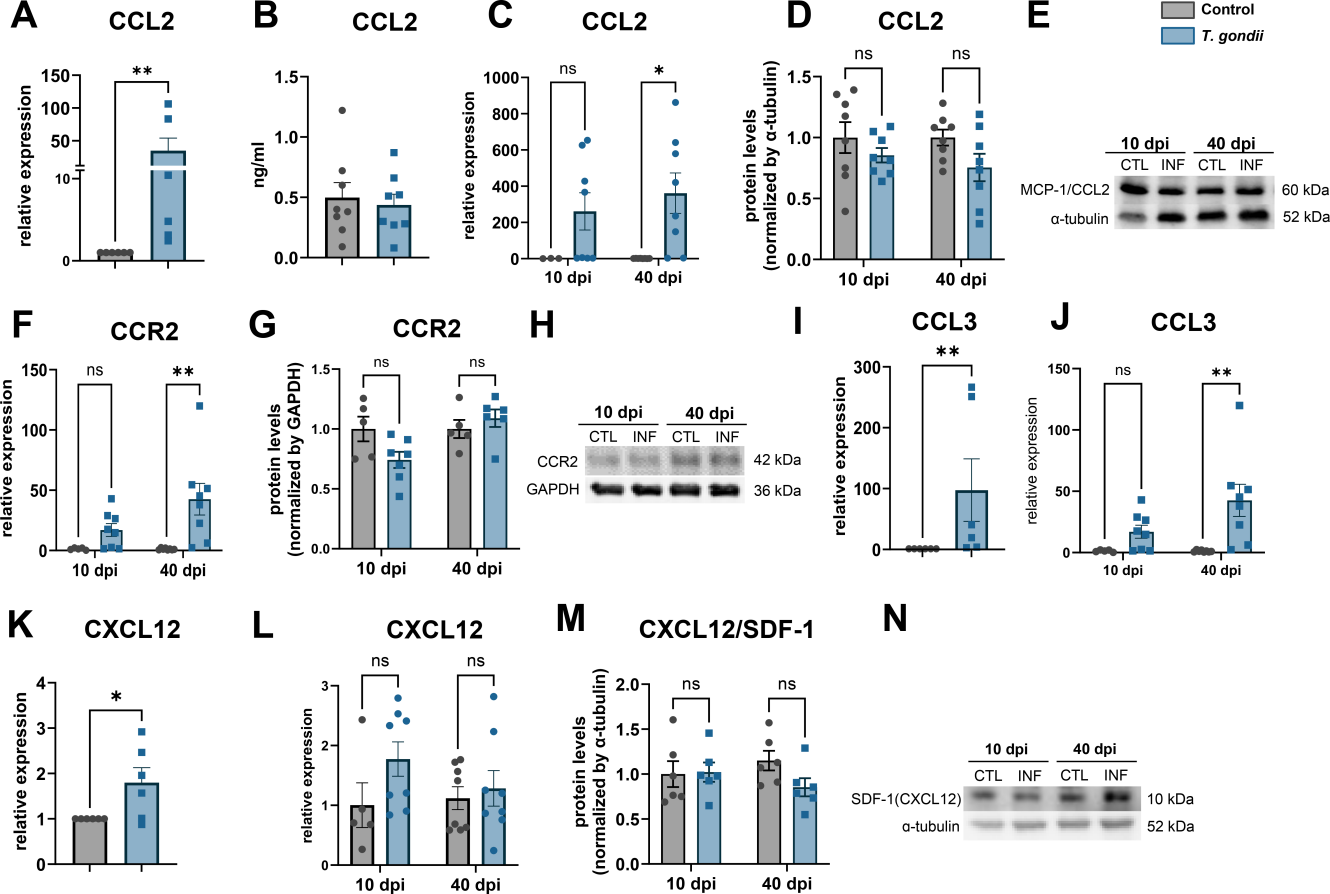
Chemokine signaling in *T. gondii* infected neurons and mouse brains. Three chemokines were consistently detected in nCM: CCL2 (MCP-1), CCL3 (MIP-1α), and CXCL12 (SDF-1). *Ccl2* expression was increased in *T. gondii*-infected neurons as shown by RT-qPCR (**A**) although no changes to *CCL-2 secretion* were found in nCM as revealed by ELISA (**B**). In mouse brain cortices, *Ccl2* expression was increased at 40 dpi, as shown by RT-qPCR (**C**), but not at the protein level (**D and E**). Similarly, CCL2 receptor (CCR2) was upregulated at 40 dpi at the mRNA (**F**), but not at the protein level (**G and H**). *Ccl3* gene expression was increased in infected neuronal cultures (**I**) and in infected brain cortices at 10 and 40 dpi (**J**). *Cxcl12* (**K**) gene expression was increased in infected neuronal cultures, but not in cortex samples, as shown by RT-qPCR (**L**) and western blotting (**M and N**). *P < 0.05, **P < 0.01, Unpaired Student’s *t*-test in (**A, B, I, and K**); two-way ANOVA with Bonferroni post-test in (**C–G**),(**J, L, and M**). Each dot in graphs in (**A, B, I, and K**) corresponds to independent neuronal culture preparation; in (**C–G, J**) and (**L and M**), to individual mouse cortices, from at least two rounds of infection. (**E, H, and N**): representative blots for CCL2, CCR2, and CCL12, respectively.

Based on the initial characterization of neuronal-derived factors that could modulate neurovascular response, we sought to further validate targets and pathways that could be triggered by a latent *T. gondii* infection and reprogram neuronal production of neuroinflammatory and neurovascular signaling. We investigated chemokines *Ccl2*, *Ccl3,* and *Cxcl12* gene expression in cortical neuron primary cultures 7 dpi. *Ccl2* transcripts were increased by 35-fold in infected neurons *in vitro* (Fig. 5A) although no significant changes were found in secreted CCL2 levels as shown by ELISA (Fig. 5B). In brain cortices, *Ccl2* expression was greatly increased both at 10 and 40 dpi (260-fold, *P* = 0.23 and 361-fold, *P* = 0.0115, respectively, Fig. 5C) although no significant changes at the protein level were observed (Fig. 5D and E). We analyzed the expression of CCL2 receptor, CCR2 and observed a similar pattern: an upregulation in gene expression at both time points (4.27-fold, *P* = 0.21 and 5.97-fold, *P* = 0.0153, respectively, Fig. 5F) with no significant changes at the protein levels (Fig. 5G and H) *in vivo*.

CCL3, also known as macrophage inflammatory protein α (MIP-1α), is a pro-inflammatory chemokine that plays an important role in CNS inflammation. *Ccl3* transcripts were significantly increased in infected neurons *in vitro* (97.5-fold, *P* = 0.0022, Fig. 5I) and in brain cortices at 10 and 40 dpi (16.9-fold, *P* = 0.4 and 42.5-fold, *P* = 0.0012, respectively, Fig. 5J).

CXCL12 one of the chemokines identified in our neuronal secretome was also assessed in our system. Although *Cxcl12* transcripts were significantly increased in infected neurons *in vitro* (1.8-fold, *P* = 0.035, Fig. 5K), no changes to CXCL12 mRNA and protein levels were observed *in vivo* (Fig. 5L and M).

**Fig 6:**
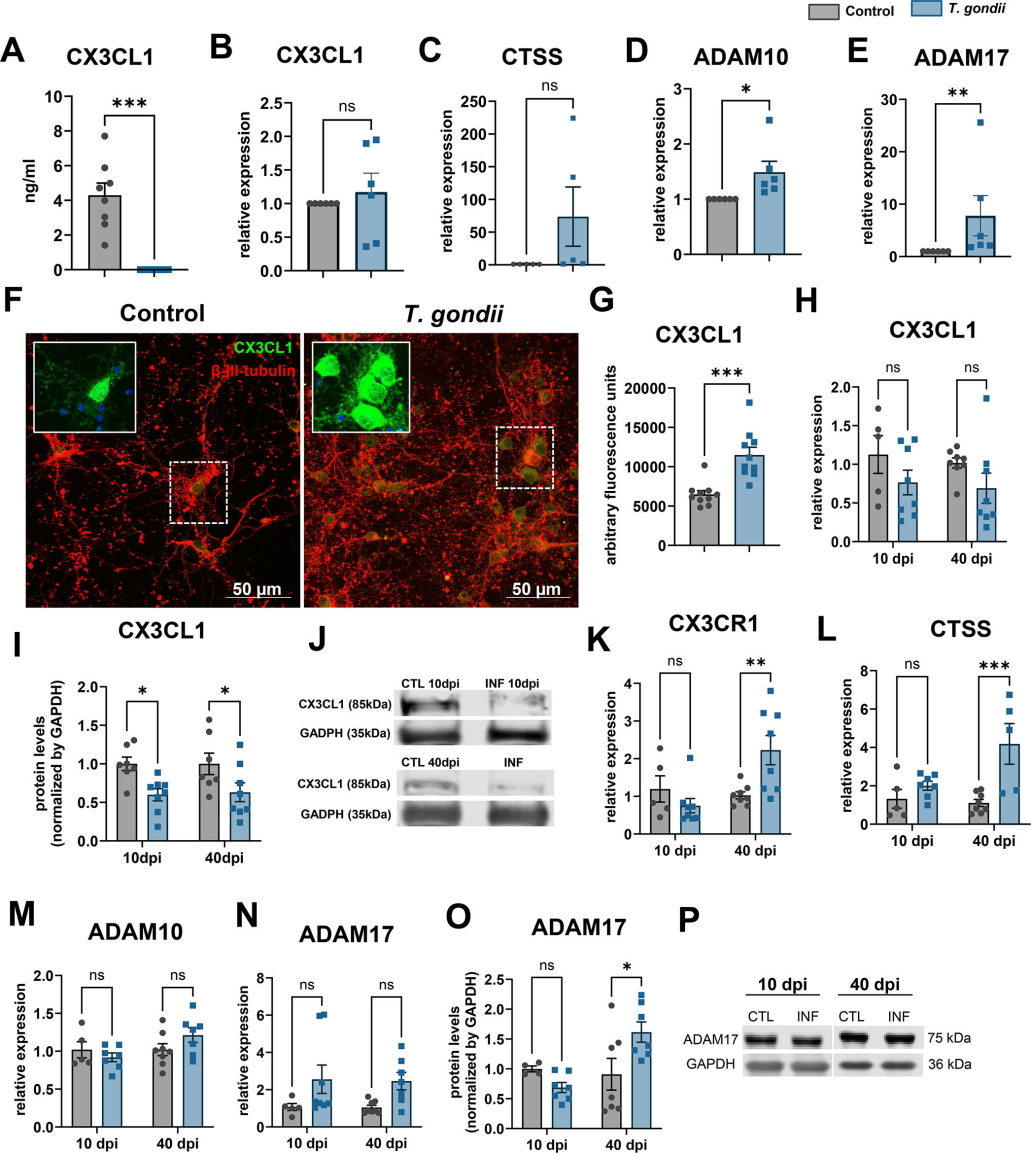
CX3CL1/Fractalkine pathway is disrupted by *T. gondii* latent infection in neurons. Fractalkine was consistently reduced in infected nCMs as shown by mini-proteome assays. *Cx3cl1* gene secretion was drastically reduced in infected cultures, as shown by ELISA (**A**) although no changes to gene expression were observed (**B**). Cathepsin S (*Ctss*), *Adam10,* and *Adam17*, known to be involved in fractalkine shedding, had their expression levels assessed by RT-qPCR (**C–E**). Fractalkine immunoreactivity and localization were analyzed by confocal microscopy (**F**). Neurons were stained with anti-β-III-tubulin (in red) and CX3CL1 (in green). Insets in F show CX3CL1 (in green) and DAPI (in blue) signals in higher magnification from the areas delimited by the dashed line squares. CX3CL1/fractalkine fluorescent signal was increased in *T. gondii*-infected dishes as compared to controls (**G**). CX3CL1 was decreased in brain cortices of infected mouse at 10 and 40 dpi, as shown by RT-qPCR (**H**) and western blotting (**I and J**), whereas *Cx3cr1* and *Ctss* transcripts were increased at 40 dpi (**K and L**). ADAM10 (**M**) and ADAM17 (**N**) had no change to mRNA levels, but ADAM17 protein levels were increased at 40 dpi (**O and P**). (**J and P**) show representative blots for CX3CL1 and ADAM17, respectively. *P < 0.05, **P < 0.01, ***P < 0.001. Unpaired Student’s *t*-test in (**A–E and G**); two-way ANOVA with Bonferroni post-test in (**H–O**). Each dot in graphs in (**A–E**) corresponds to independent neuronal culture preparation; in (**G**), to microscopic fields (with 20× objective) from two independent cultures; in (**H–O**), to individual mouse cortices, from at least two rounds of infection. Scale bars: 50 µm.

Finally, CX3CL1/Fractalkine was consistently decreased in *T. gondii*-infected nCMs, as revealed by mini-proteome assays (Fig. 2). Fractalkine is a chemokine produced primarily by neurons that binds to its cognate receptor CX3CR1, expressed in microglia (39–41). Fractalkine is a membrane-anchored protein that can be released near the transmembrane domain and shed to the extracellular space by enzymes such as ADAM10, ADAM17, and cathepsins (42, 43). Fractalkine inhibits microglial activation in different models of neurodegeneration/neuroinflammation (44, 45). We found fractalkine to be reduced from 4.29 ng/mL in control cultures, to undetectable levels in infected ones by ELISA (Fig. 6A). Although no change was verified in *Cx3cl1* and cathepsin S (*ctss*) mRNA expression *in vitro* (Fig. 6B and C), *Adam10* and *Adam17* expression had a 1.5- and 7.8-fold increase (*P* < 0.05) in infected neuronal cultures, respectively (Fig. 6D and E). Conversely, cyst-harboring β-III-tubulin-stained neurons had higher fractalkine immunoreactivity when compared to controls (Fig. 6F and G). *Cx3cl1* expression was downregulated in the cerebral cortex at 10 and 40 dpi although no statistical significance was observed (Fig. 6H), and a 85 KDa FKN band, relative to soluble FKN form, was reduced by 40% in mouse cortices at 10 and 40 dpi (Fig. 6I and J), whereas *Cx3cr1* expression was significantly increased at 40 dpi by 1.2-fold, indicative of microglial activation (Fig. 6K). Cathepsin S expression was increased by 1.7-fold in infected mouse cortices at 40 dpi (Fig. 6L), whereas *Adam10* and *Adam17* expression remained unaltered (Fig. 6M and N). ADAM17 protein content was increased by 1.6-fold at 40 dpi (Fig. 6O and P).

### Fractalkine modulates microglial polarization *in vitro*

**Fig 7:**
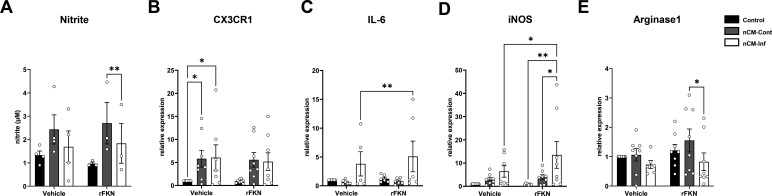
Fractalkine modulates microglial polarization upon *T. gondii* infection. BV-2 mouse microglial cell line was treated with nCM from uninfected (nCM-Cont) and *T. gondii*-infected (nCM-Inf) cultures for 24 h in the presence or absence of rFKN. Nitrite production was measured by spectrophotometry using Griess method (**A**). rFKN supplementation decreased nitrite production in nCM-Inf-treated cultures as compared to those treated with nCM-Cont. and microglial activation genes of microglia cultures treated with infected neurons supernatant. mRNA was extracted from BV-2 cultures and expression of *Cx3cr1* (**B**), *Il6* (**C**), *Nos2* (**D**) and *Arg1* (**E**) was assessed by RT-qPCR. Each dot in graphs corresponds to independent cultures. *P < 0.05, **P < 0.01, Two-way ANOVA with Bonferroni post-test.

We used nCM to treat murine microglia BV-2 cells in the presence of recombinant fractalkine (rFKN). rFKN reduced nitric oxide production BV-2 cells treated with infected nCM (nCM-Inf) as compared to cultures treated with control nCM (nCM-Cont, Fig. 7A). In order to determine the effect of nCM on microglial polarization and the role of FKN on this phenomenon, nCM-treated BV-2 cells had their RNA extracted after 24 h of treatment and expression of CX3CR1, IL-6, inducible nitric oxide synthase (iNOS), and arginase1 was studied. CX3CR1, the specific fractalkine receptor, had a significant increase in expression following treatment with either infected or uninfected nCM (Fig. 7B). The addition of rFKN to nCM led to a non-statistically significant increase in CX3CR1 expression. IL-6 expression was increased by 1.3-fold in nCM-Inf-treated BV-2 cultures when rFKN was added as compared to nCM-Inf-treated cultures (Fig. 7C). iNOS had a 6.46 ± 6.7-fold increase in nCM-Inf-treated cultures as compared to their respective controls. rFKN led to a 13.5 ± 16.2-fold increase in nCM-Inf-treated BV-2 cells (Fig. 7D). Conversely, Arginase-1 levels were significantly decreased in BV-2 cells treated with nCM-Inf in the presence of rFKN as compared to nCM-Cont (Fig. 7E).

## DISCUSSION

*T*. *gondii* infects and forms bradyzoite cysts in neurons (15, 46). Acquired *T. gondii* infection in immunocompetent mice is accompanied by neuroinflammatory response with microglial activation (47), BBB disruption (48), neurochemical imbalance (49–53), and microvascular dysfunction (9, 54), which combined, lead to neurocognitive deficits (55) and behavioral alterations (56). Additionally, treatments with sulfadiazine alone (9) or in combination with pyrimethamine (16) or ceftriaxone (51) can restore neuropathological parameters. In this work, we aimed at understanding how a latent infection in the brain could signal for a sustained neurovascular pathology.

We developed an *in vitro* model of mouse cortical neuron primary cultures and infected with tachyzoites of the Me49 strain, the same utilized in previous studies that have demonstrated such alterations *in vivo*. In our system, the tachyzoites were capable of spontaneously generating bradyzoite cysts, without inducing noticeable neuronal death. To date, this is the first description of a neuron-enriched primary culture to investigate *T. gondii* latent infection. A seminal work by Luder et al. (57) used mixed neural cultures to infect with *T. gondii* and demonstrate spontaneous tachyzoite-bradyzoite conversion in each cell population. More recently, a primary cultures of mixed neonatal rat hippocampal cells were used to generate *T. gondii* cysts (46). This model demonstrated that cysts were solely present within neurons and not in astrocytes or microglia and that bradyzoite transcriptome was similar to what was observed in freshly isolated cysts from rodent’s brains, thus indicating the relevance of this *in vitro* system.

Subsequently, we collected conditioned media from these cultures and assessed Th1/Th2 cytokine production and well as a panel of angiogenic-related molecules. Very low levels of cytokines were detected in our system, and only a slight increase in TNF production was observed. Our group has used a similar approach in the past to characterize cytokine secretion in *T. gondii*-infected cultures and described that mouse radial glial cells as well as mouse myoblasts and myotubes produce high levels of IL-6 (58, 59). Although neurons can secrete many inflammatory mediators (60) and that cytokines such as IL-4, IL-6, and TNF play an important role in synaptic physiology (61), we would expect that neuronal infection with *T. gondii* would lead to similar effects as observed in other cell types. IFN-γ stimulation was shown to degrade glutamine degradation (62) and control *T. gondii* replication in iPSC-derived human neurons (63). It is possible that any noticeable effect could have been diluted given the low MOI utilized in this study (one parasite for every 10 cells), so the levels of cytokines produced by the infected neurons could be dissipated in the media. Additionally, we have recently shown bimodal effects of *T. gondii* infection in neural progenitor cells, in which at early time points (24–72 hpi) the infection significantly affected cell proliferation, and that at later time point (96 hpi), this effect would be absent (23). This could also indicate a distinct cellular response to tachyzoite or bradyzoite infection in neural cells.

Neuronal CM was also used to profile the angiogenic factors that could be potentially altered by infection. Angiogenesis is a rapid process of blood vessel neoformation from pre-existing vessels, by sprouting, intussusceptive growth, splitting, or regression (64). Dysfunctional angiogenesis is also a hallmark of neuroinflammatory processes and correlates with BBB damage. Therefore, we used this assay as a proxy to determine which molecules are present in our cell culture system and as a guide to further investigate the ones that could be affected by the parasite. Few studies to date have looked at the role of neurons in regulating the BBB biology even though neurons are part of the NeuroVascular Unit (65), which is also comprised of BBB-forming endothelial cells, extracellular astrocytes, pericytes. We have grouped our findings into four main clusters of molecules that will be discussed below.

### Classical angiogenesis and endothelial cell modulators (VEGFA and ADAMTS1)

Angiogenesis, the formation of new blood vessels, is quiescent in the healthy adult brain and is reactivated in vascular-dependent brain pathologies such as brain vascular malformations and brain tumors. The NVU becomes deregulated in vascular-dependent brain pathologies, in which angiogenic signaling pathways become activated and form leaky, tortuous, and dysfunctional neovessels via various modes of neovascularization (66). On the contrary, rarefaction is characterized by a reduction in small vessels. The brain has little reserve capacity, so rarefaction can be deleterious. Microvascular rarefaction may be functional (a reversible reduction in perfusion) or structural (an actual anatomical loss of microvessels). A decrease in perfusion likely precedes structural rarefaction and leads to neural dysfunction and microstructural tissue damage, contributing to neurodegeneration and macrostructural lesions (67–69). We showed that *T. gondii* infection induces functional and structural capillary rarefaction in mice (9). Rarefaction may be caused by (i) impaired angiogenesis, with capillary regression, (ii) endothelial dysfunction and apoptosis, (iii) pericyte loss and loss of shear stress by tortuosity of microvessels, and (iv) vascular blockage by microemboli (70).

Angiogenic signaling depends on the balance of anti- and pro-angiogenic factors (71, 72). While VEGF remained unaltered, we identified that ADAMTS1, thrombospondin, endothelin-1, PDGFAA, and CCN family members were differentially secreted/expressed in *T. gondii*-infected neurons.

ADAMTS1 is a potent anti-angiogenic metalloprotease, which blocks VEGFR2 phosphorylation and suppresses endothelial cell proliferation (30). *Adamts1* expression was not affected by *T. gondii* infection *in vitro* but was increased at 40 dpi in infected mice cortices. ADAMTS1 is also expressed by astrocytes and microglial cells (73) and can cleave aggrecan 1, one of the most common classes of extracellular matrix chondroitin sulfate proteoglycans in the CNS (74, 75). In fact, aggrecan1 is present in the perineuronal nets, which are also known to be disturbed following *T. gondii* acquired infection in mice (76), which provides another evidence by which the parasite affects neurobiology.

TSP-1 is a matricellular anti-angiogenic protein (77), known to induce endothelial cell cycle arrest and senescence (78), inhibit MMP9 activity and suppress VEGF release from the extracellular matrix (79). TSP-1 also binds VEGF and supports its clearance through a lipoprotein receptor-related protein (LRP)-dependent mechanism (80). *Tsp1* transcripts are markedly increased in infected neuronal cultures and *in vivo* in the mouse cortex at 40 dpi. Additionally, TSP-1 is a known activator of TGF-β1 activation (81), and TGF-β1 is crucial for BMEC biology (82). Accordingly, TGF-β1 transcripts were found to be increased in mouse cortices at 40 dpi. In the brain, TGF-β modulates macrophage and microglial responses to *T. gondii* acquired infection (83, 84) and is upregulated by a dense granule protein (85). These findings indicate that angiogenic modulation could be a byproduct of immunomodulatory effects of the inflammatory process.

Another molecule that we identified was Endothelin-1 (ET-1), a potent vasoconstrictor, that regulates vascular tonus through pericyte modulation, which, in turn, contributes to blood flow dysregulation and BBB breakdown (35, 86). Interestingly, ET-1-dependent vasoconstriction was described in a mouse model of cerebral malaria (87), and we hypothesized that this molecule could also be involved in cerebrovascular abnormalities observed in the acquired toxoplasmosis mouse model. *Edn1* mRNA expression was increased in the mouse brain cortices at 40 dpi although no changes were found in ET-1 protein levels. However, circulating ET-1 levels are also known to play a role in cerebral microcirculation regulation (88) and, therefore, should be considered in future studies.

### Endothelial-regulating growth factors (IGFBP-2, -3, -9, Cyr61, and PDGF)

Cellular communication network (CCN) protein family are matricellular proteins dynamically expressed in development and injury and have cell regulatory functions (89, 90). CCN proteins comprise four domains which have homology to insulin-like growth factor binding proteins (IGFBPs), von Willebrand factor type C repeat (VWC), thrombospondin type I repeat (TSP1), and a carboxy-terminal domain (CT) containing a cystine knot. Members of this family are CCN1 (Cyr61), CCN2 (CTGF), CCN3 (NOV), CCN4 (WISP-1), CCN5 (WISP-2), and CCN6 (WISP-3).

The IGFBP family controls the availability, transport, and localization of insulin-like growth factor-I (IGF-I) (91). IGFBP-2 was the most abundant analyte found in neuronal CMs, and it is known to interact closely with ECM glycosaminoglycans to facilitate cell movement but also regulate neuronal outgrowth, CSF production, and neuronal rescue or repair in distinct types of insults (92). Even though we found considerable levels of these proteins in nCM, no overall changes were observed in brains of infected mice. Our study was limited by the fact that the RT-qPCR analyses of brain cortices do not distinguish between which cell types are producing these transcripts. Moreover, matricellular proteins are usually regulated at the post-translational level and can acquire activated forms upon cleavage. Therefore, immunohistochemical analyses are desirable to better distinguish the roles of CCN and/or IGFBP proteins in neuronal cells following *T. gondii* infection and their role in regulating microcirculatory abnormalities.

Recent studies using human iPSC-derived neurons have shown that *T. gondii can* establish latent infection and modulate host gene expression, including pathways related to neuroinflammation, synaptic function, and metabolism (63, 93). These findings closely align with our murine data, particularly regarding the upregulation of IGFBPs and inflammatory mediators, reinforcing the translational relevance of our model and suggesting conserved host responses across species.

### Chemoattractants (CCL2, CCL3, and CXCL12)

Chemokines are a family of chemoattractant cytokines characterized by their unique ability to both recruit and activate a variety of cell types. Although most commonly associated with regulation of peripheral immune cell attraction in response to physiological and pathological stimuli, chemokines can also be expressed in the CNS during development, synaptic transmission, and neuroinflammation, through microglial and astroglial activation (94). Herein, four chemokines were found in neuronal CMs: CCL2, CCL3, CXCL12, and CX3CL1.

CCL2 was recently shown to be important in controlling *T. gondii* expansion in the mouse brain (95), but its expression was mainly attributed to astrocytes in the chronic infection, whereas CCL2 receptor CCR2 is gradually increased in the brain parenchyma over infection time in mice (96). Accordingly, our results showed that although *Ccl2* expression was highly upregulated in infected neurons *in vitro*, such increase was not translated to higher cytokine secretion levels.

Neuronal CCL3 production in response to *T. gondii* infection was shown previously to be even potentiated when cultures were treated with IFN-γ and/or TNF-α (97). Previous studies have demonstrated that RANTES (CCL5) and MIP-1 (CCL4) transcripts are transiently increased in mouse brains following *T. gondii* infection (98). Similar patterns were described for chemokine receptors CXCR4, CXCR5, CCR7, and CCR8. Further studies are needed to determine the effect of CCL3 on Toxoplasma-induced neuroinflammatory responses and the role of cyst-bearing neurons in this response.

Although neuronal CXCL12 transcripts were increased in *T. gondii*-infected cultures, no changes were observed in infected brain cortices *in vivo*.

It is important to note that discrepancies between transcript levels and corresponding protein abundance, as observed for CCL2 and ET-1 in our study, are commonly reported in the literature (99, 100). Such divergences may result from post-transcriptional regulation, translational efficiency, differential protein stability, or limited detection of compartmentalized secretion. In the case of secreted mediators like chemokines and vasoactive peptides, these regulatory layers may significantly modulate local protein availability despite robust mRNA upregulation. Future studies using translational profiling or single-cell proteomics may help clarify these expression dynamics in the context of *T. gondii* infection.

### Fractalkine signaling pathway

Fractalkine is constitutively expressed at high levels by neurons, mainly in forebrain structures such as the hippocampus, amygdala, cerebral cortex, striatum, and thalamus (101). Fractalkine exists as a membrane-bound protein and as a soluble factor, released upon cleavage (102). In the brain, fractalkine is constitutively expressed by neurons, while CX3CL1 receptor (CX3CR1) is predominantly expressed by microglia (103).

We determined that *T. gondii* leads not only to a marked decrease in fractalkine secretion *in vitro* and *in vivo*, but also to a significant accumulation of its membrane-bound portion in infected cells, whereas CX3CR1 mRNA is increased at 40 dpi in infected mouse brain cortices, consistent with increased microglial activation, as previously shown by our group (9) and others (46, 104, 105).

ADAM-10, -17, and cathepsin S (CTSS) are known inducers of fractalkine cleavage (106, 107), and their mRNA expression was to some extent increased both *in vitro* and *in vivo*. Moreover, ADAMs can cleave the TNF precursor, leading to the release of its soluble form (108), and similar effect is observed for other molecules, including IL-6 (109). Herein, TNF was increased in infected cultures and is also known to be increased in *T. gondii*-infected mouse brains (110). Fractalkine cleavage and shedding from the membrane was reduced, even though these enzymes were up-regulated, suggesting that ADAMs could be mobilized to TNF cleavage in response to infection. In line with this observation, knock-down of ADAM10, but not of ADAM17, reduced FKN shedding in human brain microvascular endothelial cells (111). Future studies will be necessary to determine whether *T. gondii* infection could be inhibiting their enzymatic activity and, thus, restricting FKN release.

FKN release by neurons signals to microglia and induces a resting state (112). Conversely, administration of recombinant fractalkine or transduction with AAV vectors in neurodegeneration models, including Alzheimer’s (113, 114) and traumatic brain injury (115) restored microglial reactivity and reduced cognitive damages (116). We hypothesized that reduced fractalkine release by infected neurons could be the underlying mechanism of the sustained neuroinflammatory response in acquired toxoplasmosis model. We treated BV-2 microglial cell lines with nCM with or without the addition of recombinant FKN and observed a shift from a M2 to a M1 type of polarization, as shown by the increased iNOS and decreased Arg1 expression. Higher iNOS expression was correlated with *T. gondii* killing by macrophages (117) and highlights the immunomodulatory role of FKN in Toxoplasma neuropathology. Although instigating, these experiments will be repeated in future studies with primary microglial cell cultures.

### Conclusions

Herein, we describe a mouse neuronal primary cortical culture model that upon infection with *T. gondii* tachyzoites form bradyzoite cysts. The family of anti-angiogenic proteins ADAMTS1 and TSP-1 are increased after infection and could be involved in the capillary rarefaction observed in infected mice. Infection impairs the fractalkine shedding from neuronal membrane, thereby contributing to microglial activation. Fractalkine could be a potential target to modulate neuroinflammatory responses in acquired toxoplasmosis model and reduce neuronal loss and neurocognitive/behavioral abnormalities observed in infected animals.

## Data Availability

The data sets generated during and/or analyzed during the current study are available from the corresponding author on reasonable request.

## SUPPLEMENTAL MATERIAL

Figure S1 spectrum.01074-25-s0001.tif CBA analysis of neuronal conditional medium.

Supplemental legend spectrum.01074-25-s0002.docx Legend for Fig. S1.

ASM does not own the copyrights to Supplemental Material that may be linked to, or accessed through, an article. The authors have granted ASM a non-exclusive, world-wide license to publish the Supplemental Material files. Please contact the corresponding author directly for reuse.
