# Supplementary figures and images for: Toxoplasma gondii impairs CX3CL1/fractalkine shedding from mouse cortical neurons, leading to microglia activation
Source: Microbiol Spectr. 2025 Aug 13;13(10):e01074-25. doi: 10.1128/spectrum.01074-25 (PMC12502563; doi:10.1128/spectrum.01074-25)

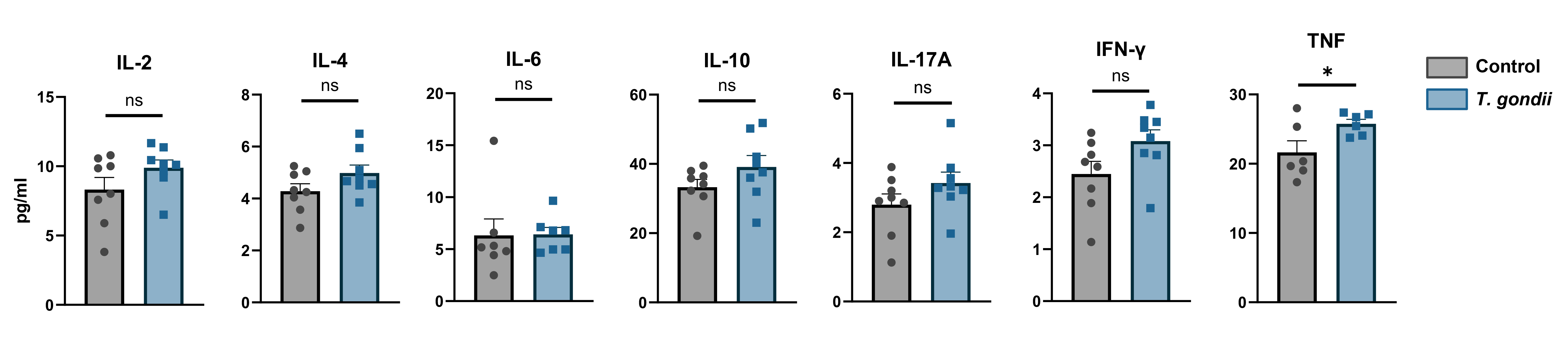

Supplement: Figure S1 — CBA analysis of neuronal conditional medium. [file spectrum.01074-25-s0001.tif]
