# Supplementary material for: Toxoplasma gondii impairs CX3CL1/fractalkine shedding from mouse cortical neurons, leading to microglia activation
Source: Microbiol Spectr. 2025 Aug 13;13(10):e01074-25. doi: 10.1128/spectrum.01074-25 (PMC12502563; doi:10.1128/spectrum.01074-25)
Supplement: Supplemental legend — Legend for Fig. S1. [file spectrum.01074-25-s0002.docx]

**Supplementary Figure S1: Cytokine production in cortical neuron cultures following *T. gondii*.** Neuronal CM were analyzed using a cytometric bead array (CBA) for Th1/2/17 responses. Levels (in pg/ml) of IL-2, IL-4, IL-6, IL-10, IL-17A, INF-γ and TNF in the supernatant of control and infected cultures. Each dot in graphs corresponds to an independent culture. Bars represent error ± standard error of the mean. *: p<0.05, Unpaired t-test.
